# Karanjin as a Neuroprotective Agent: Mitigating Amyloid Beta‐Induced Toxicity in Alzheimer's Disease Models.

**DOI:** 10.1002/alz70859_102464

**Published:** 2025-12-25

**Authors:** Manas Chakraborty, Kunal Dhiman, Veer Gupta

**Affiliations:** ^1^ Deakin University, Waurn Ponds, VIC Australia

## Abstract

**Background:**

Effective therapies for early‐stage cognitive impairment and Alzheimer’s disease (AD) remain scarce. Due to the complex nature of AD, treatment strategies must target specific biochemical pathways associated with the disease. Considering the pivotal role of metabolic disruptions in cognitive decline, this study aimed to explore alternative disease‐modifying agents, such as phytochemicals, which could influence multiple metabolic pathways. This study identified alterations in cellular bioenergetics and viability resulting from amyloid‐induced damage in cellular models. Furthermore, it evaluated the therapeutic potential of karanjin, a bioactive flavonoid, in mitigating these changes. Known for its potent antioxidant and anti‐inflammatory properties, karanjin improved cell viability and cellurlar energetics—two critical factors in the progression of neurodegeneration in AD.

**Method:**

The BE(2)‐M17 cells were employed to establish an in vitro model of AD. The cells were differentiated into mature human neurons using 10 µM retinoic acid. Before treatment with amyloid beta 1‐42 (20 µM) for 24 hours, the cells were pre‐treated with varying concentrations of karanjin (0.1 µM, 0.5 µM, 1 µM, 5 µM, and 10 µM) for 24 hours. To assess the impact of karanjin pre‐treatment on cell viability and ATP production, a Cell Titer Glo assay was conducted. Total ROS assays were performed to evaluate cellular responses, and the MTT assay was used to measure mitochondrial activity.

**Result:**

Pretreatment with karanjin at varying concentrations influenced cellular responses to 20 μM Aβ‐induced toxicity. Lower concentrations of karanjin (0.5 μM and 1 μM) effectively mitigated the reduction in cell viability caused by Aβ, with 1 μM showing the most significant improvement (*p* = 0.0379). ROS levels, measured via a luminescence assay for cellular H₂O₂, were reduced in cells pretreated with 1 μM karanjin (*p* = 0.047), highlighting its protective role against Aβ‐induced oxidative stress. Additionally, the MTT assay demonstrated that karanjin effectively preserved mitochondrial function by countering Aβ‐induced dysfunction.

**Conclusion:**

This study underscores the therapeutic potential of karanjin in mitigating amyloid beta‐induced cellular toxicity. At 1 μM, karanjin exhibited significant improvements in cell viability, oxidative stress reduction, and mitochondrial function, making it a promising candidate for targeting key mechanisms in neurodegenerative diseases like Alzheimer’s.

